# Design and Optimization of UAV Aerial Recovery System Based on Cable-Driven Parallel Robot

**DOI:** 10.3390/biomimetics9020111

**Published:** 2024-02-14

**Authors:** Jun Wu, Yizhang Sun, Honghao Yue, Junyi Yang, Fei Yang, Yong Zhao

**Affiliations:** 1 School of Mechatronics Engineering, Harbin Institute of Technology, Harbin 150080, China; 2 Aircraft Overall Design Department, Beijing Institute of Space Long March Vehicle, Beijing 100076, China

**Keywords:** UAV aerial recovery, cable-driven parallel robot, spatial cable model, error analysis, multi-objective optimization

## Abstract

Aerial recovery and redeployment can effectively increase the operating radius and the endurance of unmanned aerial vehicles (UAVs). However, the challenge lies in the effect of the aerodynamic force on the recovery system, and the existing road-based and sea-based UAV recovery methods are no longer applicable. Inspired by the predatory behavior of net-casting spiders, this study introduces a cable-driven parallel robot (CDPR) for UAV aerial recovery, which utilizes an end-effector camera to detect the UAV’s flight trajectory, and the CDPR dynamically adjusts its spatial position to intercept and recover the UAV. This paper establishes a comprehensive cable model, simultaneously considering the elasticity, mass, and aerodynamic force, and the static equilibrium equation for the CDPR is derived. The effects of the aerodynamic force and cable tension on the spatial configuration of the cable are analyzed. Numerical computations yield the CDPR’s end-effector position error and cable-driven power consumption at discrete spatial points, and the results show that the position error decreases but the power consumption increases with the increase in the cable tension lower limit (CTLL). To improve the comprehensive performance of the recovery system, a multi-objective optimization method is proposed, considering the error distribution, power consumption distribution, and safety distance. The optimized CTLL and interception space position coordinates are determined through simulation, and comparative analysis with the initial condition indicates an 83% reduction in error, a 62.3% decrease in power consumption, and a 1.2 m increase in safety distance. This paper proposes a new design for a UAV aerial recovery system, and the analysis lays the groundwork for future research.

## 1. Introduction

UAVs involve a convergence of various disciplines, including aerospace engineering, computer science, robotics, and remote sensing [1]. In recent years, there has been a surge in research efforts in the field of UAVs, with various areas of study being explored. Antennas [2,3], aircraft detection [4,5], control [6], and trajectories [7] have emerged as the most prominent research directions [8]. Moreover, research on human–UAV interaction [9], swarm behavior [10], environmental sensing [11], safety and reliability [12], and application-specific development [13] has also garnered significant attention [14]. With the development of UAV technology, UAVs have become a high-profile technological innovation in the military field [15], and the advantages of swarm UAVs in combat have become increasingly prominent. The concept of the aerial recovery of UAVs has been proposed, which greatly expands the operating radius and effective working time of UAV missions, and enables UAVs equipped with high-performance payloads to be rapidly reused. However, commonly used recovery methods for road-based and sea-based operations, such as parachute landing [16], flexible wire arresting [17], airbag landing [18], and glide landing [19], have limited deployment ranges and require complex logistical and resource arrangements. These factors hinder the rapid recovery and utilization of UAVs. Additionally, air-based recovery operations conducted in an airflow field environment pose a disadvantage due to the additional aerodynamic force, further limiting the application of existing recovery methods in the aerial domain. Currently, only the flexible towed cable–drogue scheme has been proposed for the aerial recovery of UAVs. Similar to the hose scheme for aerial refueling, the UAV autonomously approaches the drogue using its onboard camera for position recognition, and then the UAV is captured and towed into the cargo bay of the carrier aircraft using a winch [20]. This approach is exemplified by DARPA’s “Gremlins” project, as described in references [21,22].

**Figure 1:**
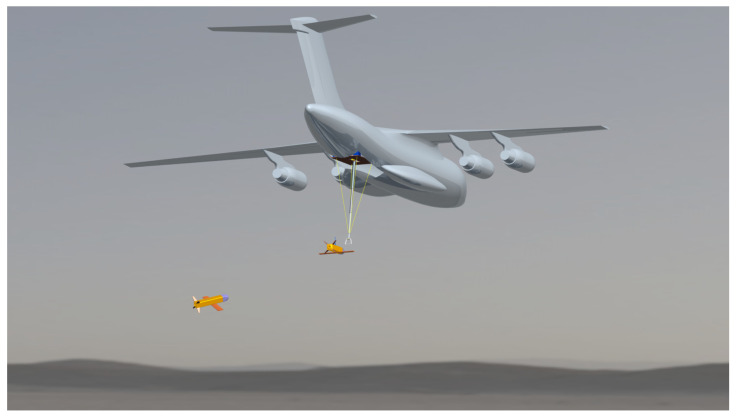
The UAV aerial recovery scenario.

However, the flexible cable–drogue towing scheme encounters challenges due to the head waves effect and the interactions between the drogue and the airflow field generated by the UAV. To address these issues, we propose an alternative solution called the “UAV Aerial Recovery Platform”. This platform is equipped with capabilities for the active detection, interception, capture, and towing of UAVs. This paper introduces the application of a CDPR for the UAV aerial recovery platform. The aerial recovery scenario is depicted in Figure 1.

The structure of a CDPR is simple and exhibits low inertia. It inherits the large working space, high load capacity, and dynamic performance typical of parallel mechanisms [23]. Existing applications of CDPRs are primarily concentrated in areas such as handling and assembly [24,25,26], 3D printing [27,28,29], medical rehabilitation [30,31], and radio telescopes [32,33]. According to the current literature, there has been no application in the field of UAV aerial recovery. This opens a potential avenue for future research and development.

Due to the varying structures of CDPRs and their different operational environments, establishing cable models involves considering different factors. In small-scale or slow-moving CDPRs, cables are often treated as ideal, neglecting the impact of the cable mass and elasticity [27,34,35]. Some studies have taken into account the elasticity of the cables [36,37], while others have considered the cable mass [38,39]. The “sagging cable” model developed by Irvine [40] is primarily used for deriving kinematic and dynamic equations that consider both the cable mass and elasticity. This model has been successfully applied to CDPRs [26,41]. Additionally, in the study of airborne flexible towing systems, some researchers have modeled the towing cables as continuum models [42,43,44,45], typically analyzing the cable elements to construct partial differential dynamic equations. Other studies have established cable models as concentrated mass models, subdivided into concentrated mass rigid body models [46,47,48,49] and concentrated mass elastic body models [50,51,52]. These models reflect the real motion of the cables, but the equations are complex and difficult to solve for multi-cable CDPRs.

For CDPRs applied in outdoor environments, they are often subjected to wind force, impacting their performance, especially for those with large sizes and high precision requirements. Zi et al. [53] introduced inverse dynamics formulas for CDPRs considering the cable mass. They simulated the wind force on a feed cabin and used fuzzy proportional–integral control to enhance the performance against wind-induced vibrations affecting the cabin’s trajectory. However, the effect of the wind force on the cables is ignored in the study. In contrast, aerial recovery missions for UAVs, particularly those conducted at high altitudes and within high-speed airflow fields, must account for the impact of the airflow force on CDPRs. In such scenarios, the flexibility of the cables, which may bend similarly to kite lines in the wind, poses additional challenges for the design and analysis of CDPR systems. Addressing the current gap in the research regarding CDPRs in airflow environments, this paper presents a study on a UAV aerial recovery system.

UAV aerial recovery is a challenging mission, and the successful interception and capture of UAVs is an important part of the process. This paper proposes a new recovery method focused on the active detection, interception, and retrieval of UAVs using a CDPR. Our work starts with developing a cable model that incorporates the aerodynamic force, and we then derive the system’s static equilibrium equations. These equations are then utilized for performance analysis and optimization. It is important to note that the fluctuating speed of the airflow is minor compared to the flight speed of the carrier aircraft. Therefore, we assume the airflow is steady and its speed equals that of the carrier aircraft.

## 2. Design of UAV Aerial Recovery System

**Figure 2:**
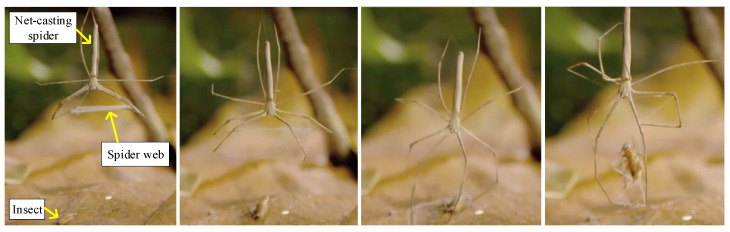
The predatory behavior of net-casting spiders.

The proposed UAV aerial recovery system in this study is inspired by the predatory behavior of net-casting spiders [54] (Deinopidae: Deinopis). Unlike other common spiders that passively wait for prey in their webs, net-casting spiders employ an active strategy of net-casting to capture insects [55,56]. When net-casting spiders hunt, they initially use their multiple legs to support the web, remaining still and patiently waiting. Upon an insect entering the capture range, they rapidly extend their long legs towards the prey (Figure 2). The spider web can adhere to the prey, capturing the insect effectively. The slender legs of spiders provide multiple advantages. On one hand, they extend the capturing range, while on the other hand, their slender nature with lower inertia enables more agile movements. During the predatory process, the eyes are employed to track the prey [55], harmonizing with the coordinated movements of multiple legs to accomplish the capture. Spider webs exhibit lightweight, flexible, and deformable features, enhancing their adaptability to the shape of the target and consequently boosting the success rate of capture.

**Figure 3:**
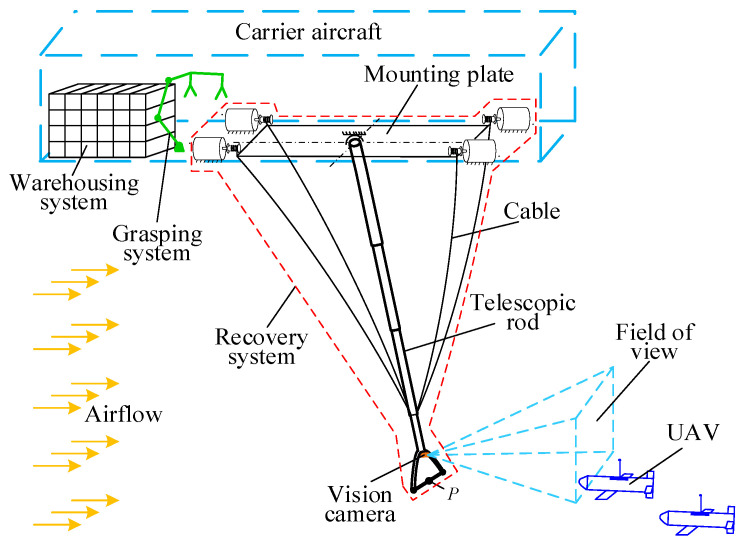
UAV aerial recovery platform.

Inspired by this predatory behavior, this paper introduces the application of a CDPR for the UAV aerial recovery platform. Figure 3 shows the system composition of the UAV aerial recovery platform. The CDPR comprises a rigid telescopic rod and four symmetrically arranged cables. The motorized telescopic rod utilizes two rotating joints between the telescopic rod and the platform, enabling pitch and yaw movements. The rigid telescopic rod plays a dual role in providing support and guidance. By adjusting the lengths of the cables and the telescopic rod, we can control the position of the CDPR’s end-effector. Moreover, the CDPR’s streamlined structure and compact volume offer reduced aerodynamic resistance in airflow, while the lower inertia enhances agility in movements. To minimize the impact on the UAV’s flight attitude, a rope–hook blocking method is used to intercept the UAV; the blocking rope is mounted at the end (marked P) of the rod and it is used to intercept the hook poking out of the top of the UAV. This method decreases the turbulence around the blocking rope, avoiding aerodynamic interference with the UAV. Additionally, the flexible blocking rope’s contact with the UAV’s rear hook prevents harsh collisions, thus reducing the risk of recovery. The CDPR employs end-effector vision cameras to detect the UAV trajectories and dynamically adjusts its position to intercept them. The recovery system is installed in the belly of the carrier aircraft. The rest of the functional systems include a grasping system and a warehousing system. The robotic arm in the grabbing system seizes and maneuvers the UAV near the nacelle to the warehousing system, which stores the recovered UAVs.

**Figure 4:**
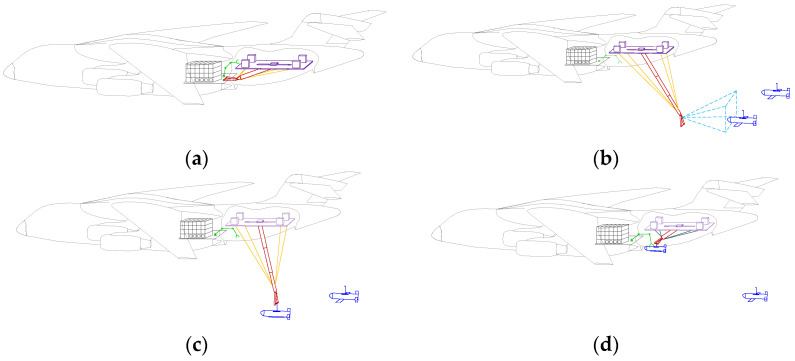
UAV aerial recovery process: (**a**) UAV aerial recovery system in the folded position; (**b**) visual detection and position adjustment; (**c**) intercept UAV; (**d**) robotic arm grabs UAV and puts it into warehousing space.

The recovery system can continuously recover multiple UAVs, and the main process of UAV aerial recovery is as follows: (1) the carrier aircraft is flying at a constant speed, the recovery system is in the folded position (Figure 4a), and the CDPR mechanical system unfolds and extends out of the carrier aircraft to stay in space underneath the carrier aircraft; (2) by detecting the position and trajectory of the target UAV from time to time with the vision camera (Figure 4b), the recovery system adjusts the position so that the blocking rope is within the hookable area of the UAV’s hook; (3) after the UAV is intercepted, the engine stops immediately and the wings are retracted (Figure 4c); (4) the CDPR tows the UAV close to the cabin, where it is transferred to the warehousing space by the robotic arm of the grasping system (Figure 4d). The above steps can be repeated for the continuous recovery of other UAVs.

## 3. Modeling and Workspace

### 3.1. Spatial Cable Modeling Considering the Elasticity, Mass, and Aerodynamic Force

**Figure 5:**
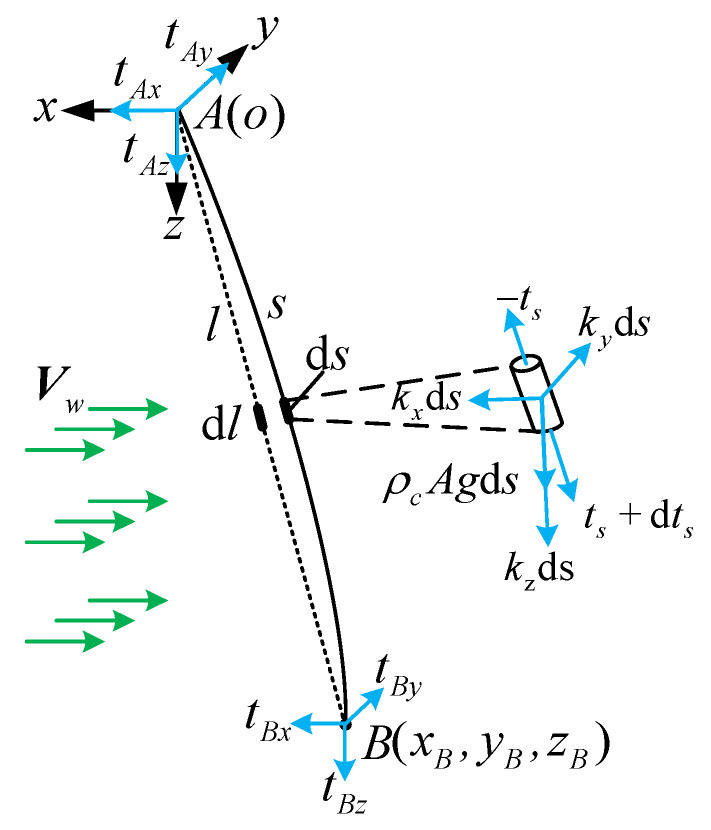
Static equilibrium force analysis of the cable.

Consider an elastic cable in static equilibrium in a steady-state airflow field with endpoint A fixed and endpoint B subjected to a force $t_{B}=(t_{\mathrm{Bx}},t_{\mathrm{By}},t_{\mathrm{Bz}})$. Establish a fixed coordinate system o-xyz at the endpoint A. The gravitational acceleration g is along the positive z-axis and the airflow velocity is expressed as $V_{w}=(v_{x},v_{y},v_{z})$. The Lagrangian coordinates of the undeformed cable and the deformed cable are l and s. The cable element ds is subjected to cross-sectional tension, aerodynamic force, and gravity. The aerodynamic force $k_{w}$ per unit length is represented by the components $k_{x}$, $k_{y}$, and $k_{z}$. Figure 5 shows the static equilibrium force analysis of the cable.

The infinitesimal element ds satisfies a geometric constraint.

(1) $${\left(ds\right)}^{2}={\left(dx\right)}^{2}+{\left(dy\right)}^{2}+{\left(dz\right)}^{2}$$

According to the principle of Hooke’s law that expresses within the elastic limit, the cable tension is expressed as:

(2) $$t_{s}=\mathrm{EA}(\frac{ds}{dl}-1)$$

where E is the elastic modulus and A is the cross-sectional area of the cable.

The tension $t_{s}$ is expressed in terms of components as:

(3) $$t_{s}=\sqrt{t_{\mathrm{sx}}^{2}+t_{\mathrm{sy}}^{2}+t_{\mathrm{sz}}^{2}}$$

The basis vectors in the coordinate system o−xyz are denoted as $\left(e_{x},e_{y},e_{z}\right)$. The tangent vector of the cable center line at the length s is denoted as $e_{t}$, the tangential airflow velocity component is $V_{\mathrm{wt}}=(V_{w}\times e_{t})e_{t}$, and the normal airflow velocity is $V_{\mathrm{wn}}=V_{w}-V_{\mathrm{wt}}$. The aerodynamic force $k_{w}$ per unit length is composed of friction resistance and pressure differential resistance, expressed as:

(4) $$k_{w}=\frac{1}{2}\rho_{a}D\pi c_{t}\left|V_{\mathrm{wt}}\right|V_{\mathrm{wt}}+\frac{1}{2}\rho_{a}Dc_{n}\left|V_{\mathrm{wn}}\right|V_{\mathrm{wn}}$$

where D is the cable diameter, $\rho_{a}$ is the air density, and $c_{t}$ and $c_{n}$ represent the friction resistance and pressure difference resistance coefficient, respectively.

The tension distribution at length s can be expressed in terms of the components of $t_{B}$ and $k_{w}$.

(5) $$\left\{\begin{array}{l} t_{\mathrm{sx}}=t_{s}\frac{dx}{ds}=t_{\mathrm{Bx}}+\int_{s}^{L_{s}}k_{x}ds\\ t_{\mathrm{sy}}=t_{s}\frac{dy}{ds}=t_{\mathrm{By}}+\int_{s}^{L_{s}}k_{y}ds\\ t_{\mathrm{sz}}=t_{s}\frac{dz}{ds}=t_{\mathrm{Bz}}+\int_{s}^{L_{s}}(k_{z}+\rho_{c}\mathrm{Ag})ds \end{array}\right.$$

Further derivation yields the following equations:

(6) $$\left\{\begin{array}{l} \frac{dx}{dl}=\frac{dx}{ds}\frac{ds}{dl}=(\frac{1}{\mathrm{EA}}+\frac{1}{t_{s}})(t_{\mathrm{Bx}}+\int_{s}^{L_{s}}k_{x}ds)\\ \frac{dy}{dl}=\frac{dy}{ds}\frac{ds}{dl}=(\frac{1}{\mathrm{EA}}+\frac{1}{t_{s}})(t_{\mathrm{By}}+\int_{s}^{L_{s}}k_{y}ds)\\ \frac{dz}{dl}=\frac{dz}{ds}\frac{ds}{dl}=(\frac{1}{\mathrm{EA}}+\frac{1}{t_{s}})(t_{\mathrm{Bz}}+\int_{s}^{L_{s}}(k_{z}+\rho_{c}\mathrm{Ag})ds) \end{array}\right.$$

The aerodynamic force acting on the cable in a steady-state airflow is related to the cable configuration. The components $k_{x}$, $k_{y}$, and $k_{z}$ are a function of s, and the integral formula in Equation (6) cannot be calculated. We consider simplifying the integral formula. Since cables can only withstand axial loads, increasing the cable tension can help to minimize the sagging and buckling caused by gravity [41]. In this paper, we discuss taut cable, which is always in a state of small bending deformation; then, the tangent direction vector of the cable can be approximated by the tension component at the endpoints B.

(7) $$e_{t}\approx (\frac{t_{\mathrm{Bx}}}{\left|t_{B}\right|},\frac{t_{\mathrm{By}}}{\left|t_{B}\right|},\frac{t_{\mathrm{Bz}}}{\left|t_{B}\right|})$$

The $e_{t}$ is independent of s, so the integral formula in Equation (6) simplifies to

(8) $$\begin{array}{l} \int_{s}^{L_{s}}k_{x}ds\approx k_{x}(L_{0}-l)\\ \int_{s}^{L_{s}}k_{y}ds\approx k_{y}(L_{0}-l)\\ \int_{s}^{L_{s}}(k_{z}+\rho_{c}\mathrm{Ag})ds\approx (k_{z}+\rho_{c}\mathrm{Ag})(L_{0}-l) \end{array}$$

where $L_{0}$ is the undeformed cable length, and $\rho_{c}$ is the cable density. After replacing the integral formula, Equation (6) becomes

(9) $$\left\{\begin{array}{l} \frac{dx}{dl}=\frac{t_{\mathrm{Bx}}+k_{x}(L_{0}-l)}{\mathrm{EA}}+\frac{t_{\mathrm{Bx}}+k_{x}(L_{0}-l)}{t_{s}}\\ \frac{dy}{dl}=\frac{t_{\mathrm{By}}+k_{y}(L_{0}-l)}{\mathrm{EA}}+\frac{t_{\mathrm{By}}+k_{y}(L_{0}-l)}{t_{s}}\\ \frac{dz}{dl}=\frac{t_{\mathrm{Bz}}+(k_{z}+\rho_{c}\mathrm{Ag})(L_{0}-l)}{\mathrm{EA}}+\frac{t_{\mathrm{Bz}}+(k_{z}+\rho_{c}\mathrm{Ag})(L_{0}-l)}{t_{s}} \end{array}\right.$$

By integrating Equation (9) with the boundary conditions x(0)=0, y(0)=0, and z(0)=0, we obtained the static profile equation using the components of l and the tension component at endpoint B.

(10) $$\left\{\begin{array}{l} x(l)=\frac{t_{\mathrm{Bx}}l}{\mathrm{EA}}+\frac{k_{x}(2L_{0}l-l^{2})}{2\mathrm{EA}}+\frac{k_{x}(b_{5}-b_{4})}{b_{3}}+(t_{\mathrm{Bx}}-\frac{b_{2}k_{x}}{2b_{3}})\Psi^{*}\\ y(l)=\frac{t_{\mathrm{By}}l}{\mathrm{EA}}+\frac{k_{y}(2L_{0}l-l^{2})}{2\mathrm{EA}}+\frac{k_{y}(b_{5}-b_{4})}{b_{3}}+(t_{\mathrm{By}}-\frac{b_{2}k_{y}}{2b_{3}})\Psi^{*}\\ z(l)=\frac{t_{\mathrm{Bz}}l}{\mathrm{EA}}+\frac{k_{z}^{*}(2L_{0}l-l^{2})}{2\mathrm{EA}}+\frac{k_{z}^{*}(b_{5}-b_{4})}{b_{3}}+(t_{\mathrm{Bz}}-\frac{b_{2}k_{z}^{*}}{2b_{3}})\Psi^{*} \end{array}\right.$$

where $k_{z}^{*}=k_{z}+\rho_{c}\mathrm{Ag}$, $\Psi^{*}=\frac{1}{\sqrt{b_{3}}}\ln(\frac{0.5b_{2}+b_{3}L_{0}+b_{5}\sqrt{b_{3}}}{0.5b_{2}+b_{3}(L_{0}-l)+b_{4}\sqrt{b_{3}}})$, $k_{x}=\frac{1}{2}\rho_{a}Dc_{n}\frac{v_{x}a_{2}-t_{\mathrm{Bx}}a_{1}}{a_{2}^{3/2}}\sqrt{a_{3}a_{2}-a_{1}^{2}}+\frac{1}{2}\rho_{a}\pi Dc_{t}\frac{t_{\mathrm{Bx}}a_{1}\left|a_{1}\right|}{a_{2}^{3/2}}$, $k_{y}=\frac{1}{2}\rho_{a}Dc_{n}\frac{v_{y}a_{2}-t_{\mathrm{By}}a_{1}}{a_{2}^{3/2}}\sqrt{a_{3}a_{2}-a_{1}^{2}}+\frac{1}{2}\rho_{a}\pi Dc_{t}\frac{t_{\mathrm{By}}a_{1}\left|a_{1}\right|}{a_{2}^{3/2}}$, $k_{z}=\frac{1}{2}\rho_{a}Dc_{n}\frac{v_{z}a_{2}-t_{\mathrm{Bz}}a_{1}}{a_{2}^{3/2}}\sqrt{a_{3}a_{2}-a_{1}^{2}}+\frac{1}{2}\rho_{a}\pi Dc_{t}\frac{t_{\mathrm{Bz}}a_{1}\left|a_{1}\right|}{a_{2}^{3/2}}$, $a_{1}=t_{\mathrm{Bx}}v_{x}+t_{\mathrm{By}}v_{y}+t_{\mathrm{Bz}}v_{z}$, $a_{3}=v_{x}^{2}+v_{y}^{2}+v_{z}^{2}$, $a_{2}=b_{1}=t_{\mathrm{Bx}}^{2}+t_{\mathrm{By}}^{2}+t_{\mathrm{Bz}}^{2}$, $b_{2}=2t_{\mathrm{By}}k_{y}+2t_{\mathrm{Bx}}k_{x}+2t_{\mathrm{Bz}}(k_{z}+\rho_{c}\mathrm{Ag})$, $b_{3}=k_{x}^{2}+k_{y}^{2}+{\left(k_{z}+\rho_{c}\mathrm{Ag}\right)}^{2}$, $b_{4}=\sqrt{b_{1}+b_{2}(L_{0}-l)+b_{3}{\left(L_{0}-l\right)}^{2}}$, $b_{5}=\sqrt{b_{1}+b_{2}L_{0}+b_{3}L_{0}^{2}}$.

If we make $l=L_{0}$, v=0 m/s, then Equation (10) degenerates to the coordinates of point B, the same as in the existing literature [57] using the elastic catenary cable model.

### 3.2. Static Equilibrium Equation

**Figure 6:**
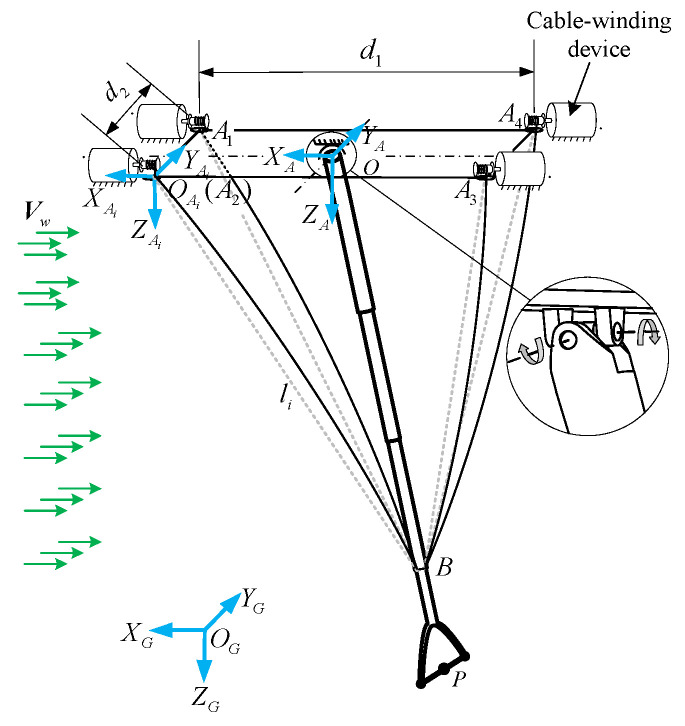
Configuration and structural parameters of CDPR.

In Figure 6, establish inertial coordinate system $O_{G}-X_{G}Y_{G}Z_{G}$ and aircraft body coordinate system $O-X_{A}Y_{A}Z_{A}$, in which the direction of $OX_{A}$ is the same as the flight direction. Establish a local coordinate system $O_{A_{i}}-X_{A_{i}}Y_{A_{i}}Z_{A_{i}}$ at the endpoint $A_{i}(i=1,2,3,4)$ of the cable, in the same direction as the body coordinate system. The endpoints $A_{i}$ are symmetrically arranged on the platform, with length and width distances of $d_{1}$ and $d_{2}$, respectively. The telescopic rod has rotational degrees of freedom in both the x and y axes. One end of each of the four cables is anchored at a point B on the telescopic rod, while the other end is connected to the cable-winding device.

**Figure 7:**
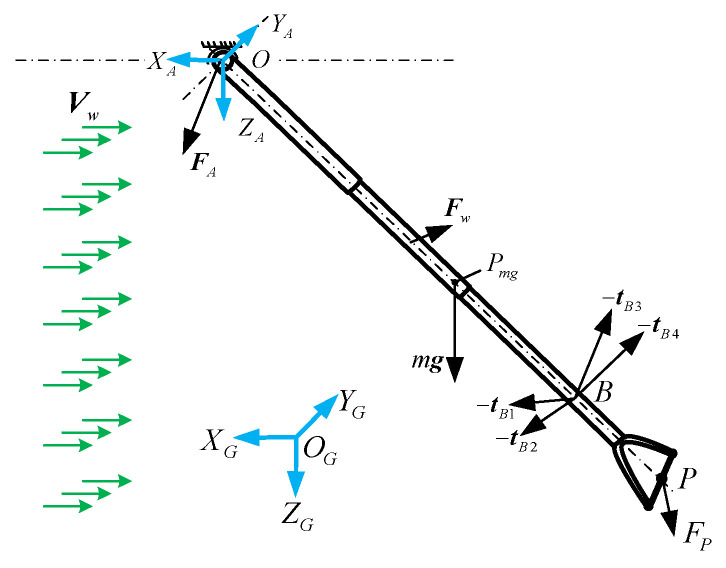
Force analysis of telescopic rod.

Figure 7 shows the force analysis of the telescopic rod, which is in static equilibrium under the tension of the cables $t_{Bi}(i=1,2,3,4)$, aerodynamic force $F_{w}$, gravity, and the platform support force $F_{A}$.

The equilibrium equations of forces and moments in static equilibrium for the telescopic rod in the body coordinate system are as follows

(11) $$\begin{array}{l} -\sum_{i=1}^{4}t_{\mathrm{Bi}}+F_{A}+F_{w}+mg+F_{P}=0\\ (\left|\vec{\mathrm{OP}}\right|-\left|\vec{\mathrm{PB}}\right|)\frac{\vec{\mathrm{OP}}}{\left|\vec{\mathrm{OP}}\right|}\times (-\sum_{i=1}^{4}t_{\mathrm{Bi}})+M_{w}+M_{\mathrm{mg}}+\vec{\mathrm{OP}}\times F_{P}=0 \end{array}$$

where $\left|\vec{\mathrm{PB}}\right|$ is a constant, and $M_{\mathrm{mg}}$ is the moment generated by the gravity of the telescopic rod at point O; it is equal to the vector product of the $\vec{OP_{\mathrm{mg}}}$ and the gravity force mg. The vector $\vec{OP_{\mathrm{mg}}}$ can be expressed as $\mu \vec{\mathrm{OP}}$, where the scale factor μ is a function of the length $\left|\vec{\mathrm{OP}}\right|$, which can be obtained by fitting the data (see Appendix A). $M_{w}$ is the aerodynamic moment of the telescopic rod with respect to point O, obtainable through fitting the finite element simulation data (see Appendix A). The external load force on the end-effector of the CDPR, denoted as $F_{P}$, is generated by the interaction between the CDPR and the UAV; its value is 0 before the UAV is captured.

The unknown variables in static balance equations are $F_{A}$, $\vec{\mathrm{OP}}$, $t_{\mathrm{Bi}}$, and $L_{i}(i=1,2,3,4)$, totaling 22 variables. The number of equations is less than the number of unknowns, and the solutions for the cable tension and cable length are not unique. Additional constraints must be introduced to the solution to ensure a unique and meaningful outcome.

### 3.3. Workspace and Interception Space

Considering the limitations of the telescopic rod length and ignoring the diameter effect, the CDPR’s reachable workspace is a hollow hemisphere. However, the actual workspace is limited by geometric constraints, which further reduce the workspace. In the case of this paper, the geometric constraints include the following:

Constraint 1: Telescopic rod length limit, expressed in terms of the components of the point P as $3.12\;m\leq \sqrt{P_{x}^{2}+P_{y}^{2}+P_{z}^{2}}\leq 6.22\;m$;

Constraint 2: Minimum distance limit between UAV and carrier aircraft; the z-axis coordinates of point P are constrained to be $P_{z}\geq S_{l}$, $S_{l}$ takes the value of 2.5 m;

Constraint 3: The permissible angle of intersection between the blocking rope and the plane of xoy is from −20° to 20°, expressed as $70{}^{\circ}\leq 90{}^{\circ}-\arctan\frac{P_{y}}{P_{z}}\leq 110{}^{\circ}$.

**Figure 8:**
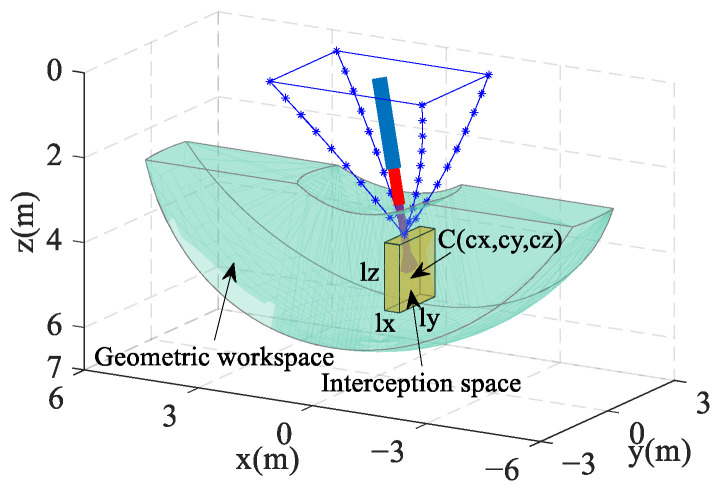
Geometric workspace and interception space.

**Figure 9:**
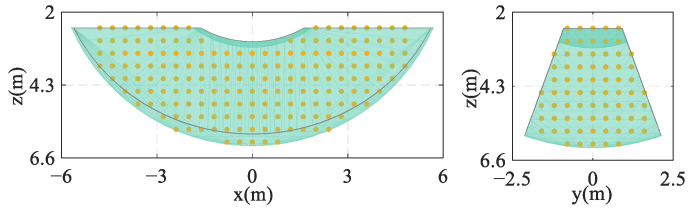
Discrete points within the workspace.

In Figure 8, the interception space is the area where the CDPR end-effector is active before the capture of the UAV, it is contained within the workspace of the CDPR, and its spatial location can be described using the coordinates of the geometric center point $C(c_{x},c_{y},c_{z})$. For ease of calculation, the CDPR workspace is discretized into scatter points with a spatial spacing of 0.4 m. The size of the interception space dimension $l_{x}\times l_{y}\times l_{z}$ is defined to be 0.4 m×0.8 m×1.2 m, and each interception space covers 24 spatially spaced points. Figure 9 gives an arrangement of the scatter points within the workspace, where the edge location scatter points cannot be accommodated by a complete interception space and are therefore ignored in the figure.

## 4. Simulation Analysis of Cable and DCPR

### 4.1. Cable Analysis

#### 4.1.1. Analysis of the Effect of Airflow on Cable Configuration

**Figure 10:**
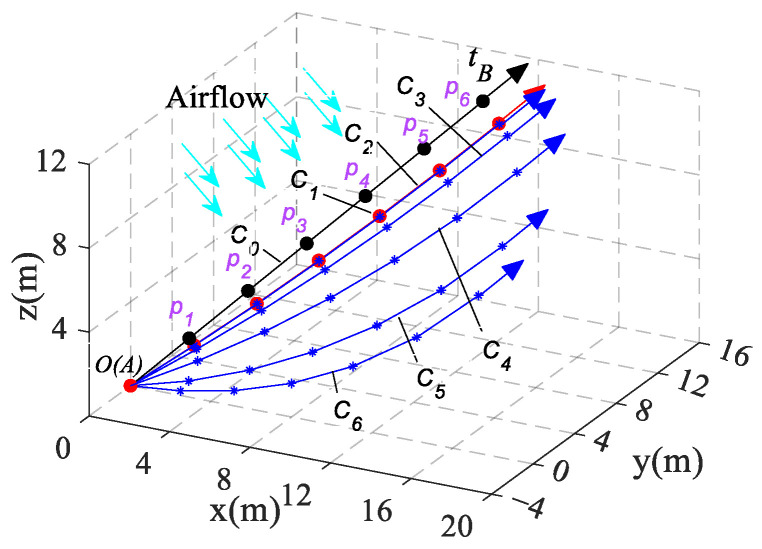
The cable spatial equilibrium configurations for different airflow velocities *v*.

In three dimensions, a cable of length 20 m is anchored at the origin A and subjected to a constant force $t_{B}=500(1/\sqrt{3},1/\sqrt{3},1/\sqrt{3})N$ at point B. The cable’s diameter D=0.01 m, density $\rho_{c}={5.16\;\mathrm{kg}/m}^{3}$, elastic modulus E=194 GPa, and acceleration of gravity $g=9.8\;m/s^{2}$ are oriented along the -z-axis. The equilibrium configuration of the cable, disregarding the effects of the mass and aerodynamic force, is denoted as $C_{0}$; it is clear that the $C_{0}$ is a straight line. The configuration considering the mass of the cable is denoted as $C_{1}$. For the analysis, the airflow vector V is chosen perpendicular to the configuration $C_{0}$, and the drag coefficient $c_{n}$ is set to 0.8. In Figure 10, the equilibrium configurations $C_{i}(i=2,3\ldots 6)$ of the cable are determined by introducing parameters into Equation (10) with various airflow velocities v.

At equal intervals along the cable length, observation points $p_{i}(i=1,2,3\ldots 6)$ are marked. The three-dimensional spatial Euclidean distance of each observation node changing from the $C_{0}$ to the $C_{1}$ is defined as $d_{i}^{01}(i=1,2,3\ldots 6)$. Similarly, the Euclidean distance of each observation node changing from the $C_{1}$ to the $C_{i}(i=2,3\ldots 6)$ is denoted as $d_{i}^{1j}(i=1,2,3\ldots 6,\;j=2,3,4\ldots 6)$. To analyze the effect of various airflow velocities on the cable configuration, a relative scale value $\frac{d_{i}^{1j}}{d_{i}^{01}}(i=1,2,3\ldots 6,\;j=1,2,3\ldots 6)$ is introduced. This value indicates the percentage change in the cable configuration due to the aerodynamic force relative to the change caused by the cable’s mass. The results of these calculations at different airflow velocities are presented in Table 1.

Figure 10 and Table 1 demonstrate that as the airflow velocity increases, the cable’s position deviates from $C_{1}$. At a lower velocity, the aerodynamic force exerts a minor influence on the cable’s spatial configuration, accounting for less than 5%, while the cable’s configuration is predominantly influenced by its gravitational force. However, at an airflow velocity of 26.4 m/s, the impact of the aerodynamic force becomes comparable to gravity, constituting approximately 102%. This signifies a critical point where the aerodynamic force becomes significant and cannot be overlooked. As the wind speed surpasses 50 m/s and beyond, the effect of the aerodynamic force exceeds 300% in comparison to gravity, becoming the predominant factor reshaping the cable’s spatial configuration.

#### 4.1.2. Analysis of the Effect of Tension on Cable Configuration

**Figure 11:**
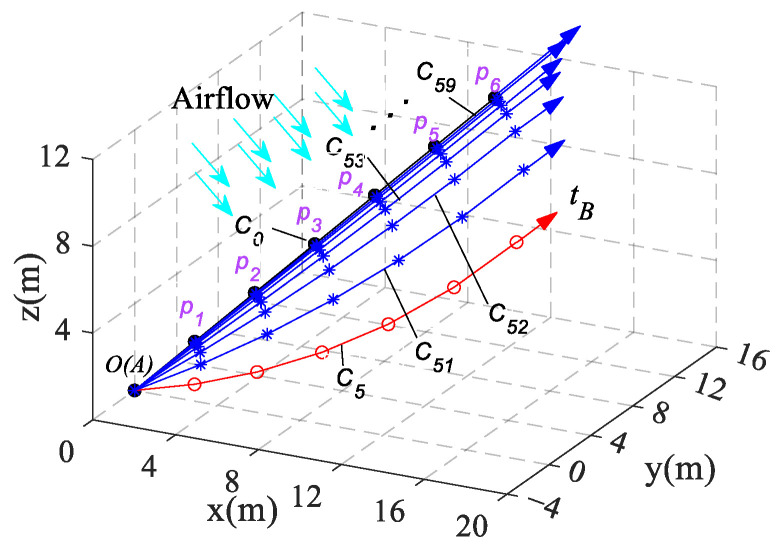
The cable spatial equilibrium configurations under different tensions.

In aerial recovery missions, it is important to consider various factors such as the mission environment characteristics and the flight capabilities of UAVs to determine the size of the carrier aircraft’s flight speed, and a speed range of 60~100 m/s may be a good choice. In this section, we take $C_{5}$ (*v* = 80 m/s) as the initial configuration and increase the tension of the cable at the endpoint B while keeping the force direction unchanged. This allows us to observe the different configurations of the cable under varying tension $t_{B}$, as illustrated in Figure 11.

We define the distance of each observation point from the $C_{5k}(k=0,1,2\ldots 5)$ to the $C_{0}$ is denoted as $d_{i}^{5k}(i=1,2,3\ldots 6\;,k=0,1,2\ldots 5)$. The calculation results for these distances are presented in Table 2.

From the data analysis, it is evident that the bending of the cable caused by the mass and aerodynamic force can be reduced by increasing the cable tension, and the equilibrium configuration is gradually approaching the straight cable. Furthermore, the data show that the effect of reducing the cable bending by increasing the cable tension is not obvious when the cable tension is large.

### 4.2. Position Error and Power Consumption Analysis in CDPR

#### 4.2.1. Position Error Analysis

The successful interception of UAVs during aerial recovery plays a crucial component in the overall recovery process. However, the presence of an end-effector position error affects the accurate interception and recovery of UAVs. Therefore, it is essential to research the end-effector error distribution of the CDPR.

The end-effector error can be defined as the Euclidean distance between the target point $P_{i}$ and the actual balance point $P^{\prime }$, and the error can be calculated using the following procedure: Firstly, the cable is modeled as a straight cable, and the cable length $l_{i}(i=1,2,3,4)$ that satisfies the geometric constraints is determined without considering the aerodynamic force and external loads. Next, by incorporating the cable length *l_i_* into the static equilibrium equation, which considers factors such as the mass and aerodynamic force, we can solve the equilibrium position $P^{\prime }$. Finally, the error is the distance between the target position $P_{i}$ and the equilibrium position $P^{\prime }$.

**Figure 12:**
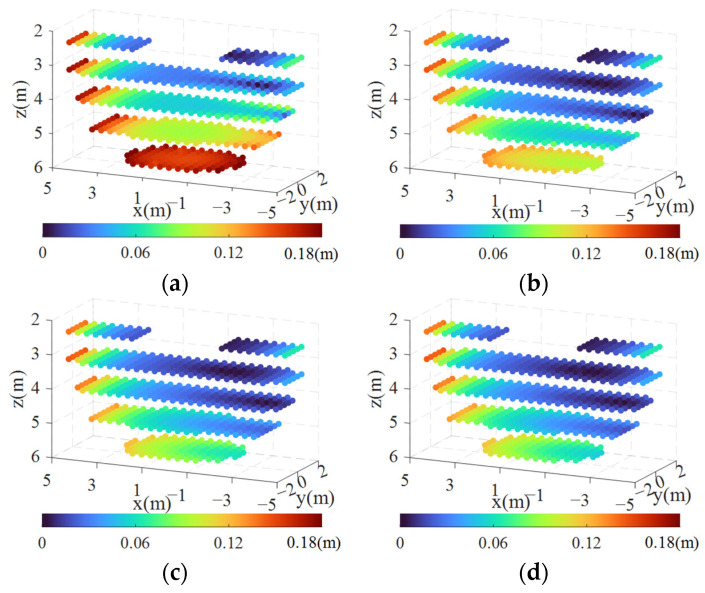
Position error distribution under different CTLL conditions: (**a**) CTLL = 0 N; (**b**) CTLL = 500 N; (**c**) CTLL = 1000 N; (**d**) CTLL = 1500 N.

However, the CDPR operates in an airflow environment where the aerodynamic force causes spatial bending in the cables, resulting in a position error at the CDPR end-effector. Previous analyses have shown that increasing the cable tension can reduce cable bending. To mitigate the CDPR end-effector error, we introduce the CTLL constraint while determining cable length $l_{i}$. This entails meeting both the geometric constraints and the force equilibrium prerequisites. Subsequently, we calculate the theoretical cable length $l_{i}^{\prime }(i=1,2,3,4)$, and incorporate $l_{i}^{\prime }$ into the equilibrium equation to derive $P_{i}^{\prime }$, and compute the position error. To minimize the computational effort, we evaluate the error at select discrete points, as illustrated in Figure 9. The structural and aerodynamic parameters for the CDPR are detailed in Table 3. Additionally, Figure 12 displays the error distribution of the CDPR under varying CTLLs. For clarity, only data from uniformly spaced sections perpendicular to the z-axis are presented in the figures.

**Figure 13:**
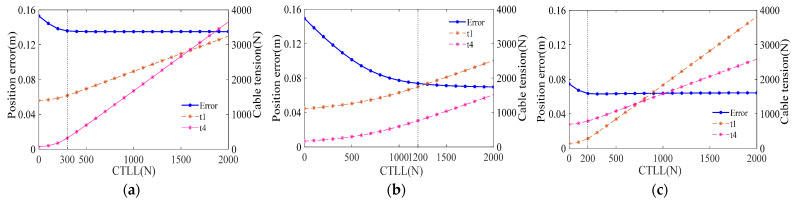
Errors and cable tensions vary with VCLL at different points: (**a**) $C_{1}(4.8,0,2.5)$; (**b**) $C_{2}(-4.8,0,2.5)$; (**c**) $C_{3}(0,0,5.7)$.

It can be observed from the four error distribution diagrams that the error is symmetrically distributed along the xoz plane, and the error decreases with the increase in the CTLL. In order to analyze more deeply, three reference points distributed at the edge of the workspace are selected, with coordinates $C_{1}(4.8,0,2.5)$, $C_{2}(-4.8,0,2.5)$, and $C_{3}(0,0,5.7)$, and the errors and cable tensions under different CTLLs are calculated, as shown in Figure 13. Due to the symmetrical arrangement of the cable, the values of the cable tension on both sides are symmetrical, and $t_{1}$ and $t_{4}$ are only drawn in the figures.

The curves indicate that the cable tension at the reference point increases with an increase in the CTLL, while the error decreases with an increase in the CTLL. However, after the CTLL reaches a certain value, increasing the VCTLL continuously does not significantly reduce the error. We can also observe from the graphical data that the error values for points $C_{1}$, $C_{2}$, and $C_{3}$ tend to remain constant once the CTLL reaches 300 N, 200 N, and 1200 N, respectively. To explain the reasons for this, the following analysis is performed.

**Figure 14:**
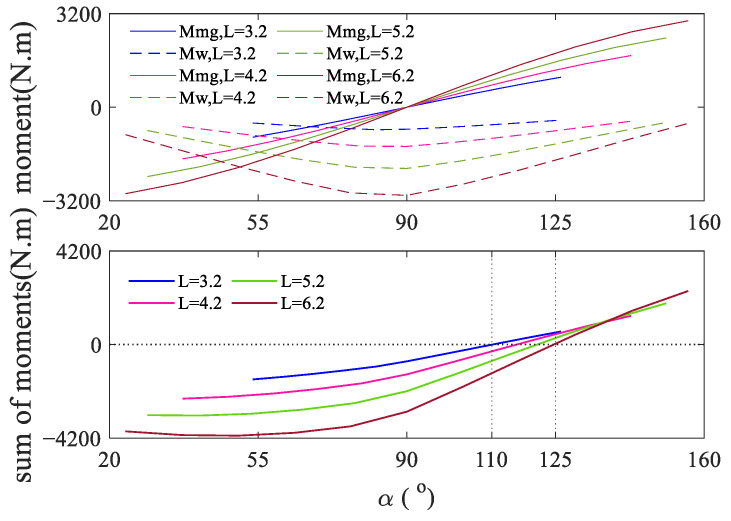
Moments on the telescopic rod.

Figure 14 illustrates the correlation between the inclination angle α of the telescopic rod in the xoz plane and the applied aerodynamic moment $M_{w}$ and gravity moment $M_{\mathrm{mg}}$ on the telescopic rod. Since the aerodynamic force and gravity are both in the *xoz* plane, $M_{w}$ and $M_{\mathrm{mg}}$ contain only y direction components, the vertical coordinates in the graph indicate the component values, and the positive and negative values represent the direction only. Overall, the values of $M_{w}$ and $M_{\mathrm{mg}}$ increase as the length of the telescopic rod increases. $M_{w}$ always acts in the −y direction; as the value of α increases, the value of moment $M_{w}$ initially rises and then decreases. This phenomenon is attributed to the angle between the telescopic rod and the airflow direction, and $M_{w}$ is maximized when the angle is around 90°. The summation curves of $M_{w}$ and $M_{\mathrm{mg}}$ are depicted in Figure 14, and it is evident that the summation function between 110° and 125° exhibits either a zero value or a smaller value, indicating a near balance between the aerodynamic moment of the telescopic rod and the gravity moment. As we understand from solving the static equilibrium equations, the cables must counterbalance the summation moment on the telescopic rod. When the summation moment is larger, the new equilibrium state deviates more from the theoretical state, resulting in a larger end-effector error. Conversely, when the summation moment is smaller or zero, the new equilibrium state is closer to the theoretical state, leading to a smaller error. Upon observing Figure 14, the summation moment value is highest when α is between $20^{\circ }∼90{}^{\circ}$ and the rod length is long, which corresponds to the windward and bottom positions in the workspace. This explains why the larger error area is distributed windward and at the bottom of the workspace, while the smaller error area is concentrated in the middle and rear areas of the workspace.

For the CDPR proposed in this paper, which is in the airflow field environment, the cables bend under the gravity and aerodynamic force, which causes the positional error at the end-effector of the CDPR, and this part of the error can be reduced by increasing the CTLL. Therefore, when the CTLL increases to a certain value, the error caused by the spatial bending of the cable becomes very small; at this time, the error in the CDPR primarily arises from the cable balancing the summation moment on the telescopic rod. However, the summation moment applied to the telescopic rod is not affected by the CTLL, and this is the reason why the error of the reference point in Figure 13 decreases with the increase in the CTLL and finally, tends to be constant.

#### 4.2.2. Power Consumption Analysis

The recovery system predicts the UAV trajectory and adjusts its position before capturing the UAV, and the entire process of intercepting and capturing is deliberately slow due to safety considerations. Consequently, the issue of power consumption deserves attention. The cable-driven power consumption of the CDPR is calculated as

(12) $$P=\sum t_{\mathrm{Ai}}v_{i},i=1,2,3,4$$

where $t_{\mathrm{Ai}}$ is the tension of the cable at the driving end, and $v_{i}$ is the driving speed of the cable. Considering that the moving direction of the end of the CDPR is arbitrary during the process of adjusting the position, it is stipulated that the average power consumption in the x, y, and z directions is adopted as the power consumption at this position. The tuning speed of the end-effector is set to be $v_{P}=0.1\;m/s$, and the cable-driven power consumption of the CDPR at any point in the workspace is expressed as

(13) $$P_{P}=\frac{\sum_{j=1}^{3}\sum_{i=1}^{4}t_{\mathrm{Ai}}v_{\mathrm{Ai}}^{j}}{3}$$

where $t_{\mathrm{Ai}}$ denotes the tension of the *i*th cable, and $v_{\mathrm{Ai}}^{j}$ represents the driving speed of the *i*th cable in the *j*th direction.

**Figure 15:**
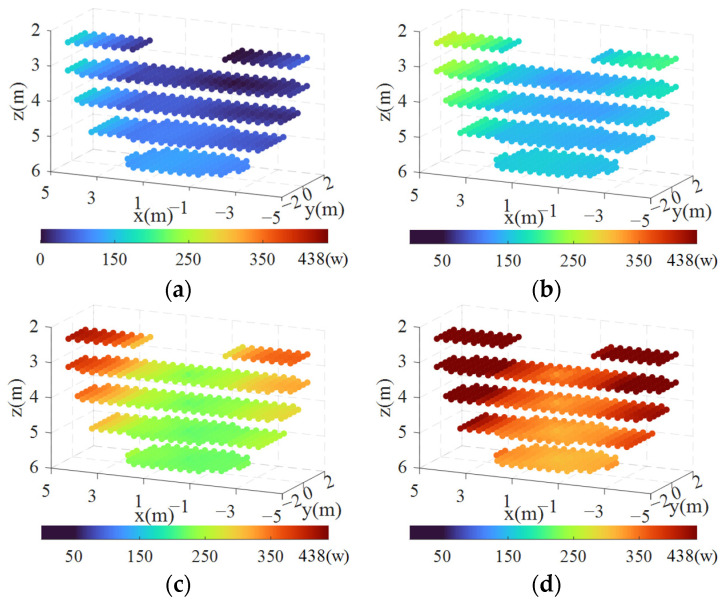
Power consumption distribution under different CTLL conditions: (**a**) CTLL = 0 N; (**b**) CTLL = 500 N; (**c**) CTLL = 1000 N; (**d**) CTLL = 1500 N.

Figure 15 displays the spatial distribution of the power consumption under various CTLL conditions. It is obvious that the power consumption shows an overall increasing trend with the increase in the CTLL. Unlike the results obtained from the error analysis, the power consumption value does not converge to a constant value as the CTLL increases. This is because the increase in the CTLL directly leads to an increase in the tension of each cable. When the CTLL is fixed, the power consumption remains relatively low in the region near the z-axis but increases in the surrounding areas, particularly at the extremities of the x-axis; this occurrence becomes more pronounced with larger values of the CTLL.

## 5. Multi-Objective Optimization of the UAV Aerial Recovery System

There are various approaches for addressing MOPs, generally categorized based on the timing of incorporating preferences from the problem operator or DM: no preference [58], a priori [59], interactive [60], or a posteriori methods [61,62]. In a posteriori methods, a set of representative Pareto-optimal solutions are obtained, allowing the DM to analyze the trade-off relationships between the objective [63]. This method is widely used in the literature to solve real problems. One of its advantages is the ability to find PFs, and this can be achieved with just one program run. The TOPSIS method, proposed by Hwang et al. [62], determines the best compromised solution, which is the one closest to the positive ideal solution and farthest from the negative ideal solution within the Pareto set. This determination is made based on objective weights and the normalization of these solutions [63]. This paper carries out an optimization analysis based on this method. Firstly, each optimization objective is normalized, then appropriate weight coefficients are selected according to the task requirements, and finally, the optimization algorithm is used to solve the problem.

For the UAV aerial recovery system, we hope that the CDPR has a good position accuracy and reasonable interception space before capturing the UAV. As previously analyzed, the position error can be reduced by increasing the CTLL, but the increase in the cable tension will lead to an increase in the power consumption of the cable drive, and the CDPR will dynamically adjust the end-effector position before capturing the UAV, which is a slow and time-consuming process. Therefore, the problem of power consumption cannot be ignored. In the case of a certain CTLL, the interception space location not only affects the end-effector position error and power consumption, but also the distance between the interception space and the carrier aircraft affects safety. We want the interception space to be away from the carrier aircraft in the z-direction to increase the recovery safety. In response to the above problems, in this paper, we optimize the CTLL and interception space location based on the CDPR end-effector position error, power consumption, and safety distance.

### 5.1. Optimization Objective Function

The CTLL and interception space location are set as optimization variables to optimize the performance of the recovery system. The objectives of the optimization are to reduce the CDPR end-effector position error, the power consumption, and increase the safety distance. We refer to the multi-objective optimization method presented in [64], which converts multiple-objective functions into a single-objective function. The objective function of the optimization problem is expressed as

(14) $$\mathrm{OBJ}=\lambda_{1}\frac{E}{E^{*}}+\lambda_{2}\frac{P}{P^{*}}+\lambda_{3}\frac{S^{*}}{S}$$

where $E^{*}$, $P^{*}$, and $S^{*}$ are the reference values of each main performance index before optimization, $\lambda_{i}$ is the weighting factor assigned to the *i*th performance index, and its distribution depends on the focus of the optimization problem. UAV recovery missions place greater emphasis on operational precision and safety; therefore, the weight factors are $\lambda_{1}=0.4$, $\lambda_{2}=0.2$, and $\lambda_{3}=0.4$. E and P denote the mean values of the position error and power consumption of the scattering points in the interception space, respectively, and *S* denotes the distance between the center of the interception space and the recovery platform. These values are calculated as follows:

(15) $$\begin{array}{l} E=\frac{\sum p_{e}^{i}}{n}(i=1,2,3\ldots n)\\ P=\frac{\sum p_{P}^{i}}{n}(i=1,2,3\ldots n)\\ S=c_{z} \end{array}$$

where n denotes the number of scatter points in the interception space, and $p_{e}^{i}$ and $p_{P}^{i}$, respectively, denote the position error and power consumption values at the location of the *i*th point in the interception space. The initial interception space is set at the front of the workspace along the direction of flight, the position coordinate is (4.6,0,3.1), and the CTLL is set to 1000 N. The calculated values of E and P are 0.124 m and 389 w, respectively, and the value of S is 3.1 m.

### 5.2. Optimization Results

The primary method for solving MOPs is typically stochastic algorithms, commonly referred to as meta-heuristic algorithms. Generally, these algorithms are classified into four main groups based on the inspiration for their development: evolution-based, physical phenomena-based, human behavior-related, and swarm-based [63]. Typical optimization methods include GA, PSO, DE, ACO, and MOSA [65]. Indeed, GAs have relatively simple and easy-to-understand basic principles, making them accessible for implementation. They often exhibit strong global search capabilities, allowing them to explore diverse solution spaces. GAs are capable of handling discrete types of variables, making them widely applicable across various domains.

The optimization problem was solved using the genetic algorithm for the purpose of finding a global solution to the optimization problem. For the genetic algorithm, set the parameters as follows: population size 50, initial population randomly generated, number of elites 5, cross progeny ratio 0.8.

**Figure 16:**
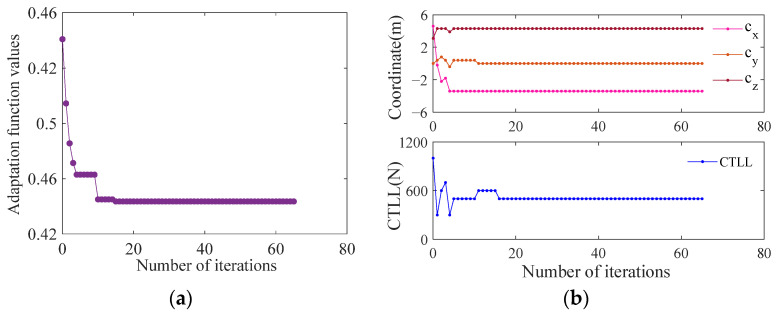
Convergence value of optimization: (**a**) adaptation function; (**b**) design variables.

Figure 16 shows the values of the design variables and the fitness function during the optimization process; it can be seen that the optimization converges after 15 iterations, and the final value of the fitness function is 0.432 and the optimization results are shown in Table 4.

The optimization results show that the interception space position mainly moves in the direction of the −x and +z axes, especially in the direction of the −x axis. Combined with the previous analysis results, the position error and power consumption of the middle and rear of the workspace are smaller than those of the other areas. This is one of the reasons why the interception space converges near this area. After optimization, the CTLL changes from 1000 N to 500 N, and the interception space position coordinate is (−3.4, 0, 4.3). The performance indexes are improved significantly, the CDPR position error is changed from 0.124 m to 0.021 m and reduced by 83%, the power consumption is changed from 389 w to 146.5 w and reduced by 62.3%, and the safety distance is increased by 1.2 m. 

## 6. Conclusions

In this paper, a new method is proposed for the UAV aerial recovery mission, wherein a CDPR is employed as the manipulator for the recovery system. The design utilizes a 3Dof CDPR with four cables towing a rigid telescopic rod to achieve UAV aerial active interception and recovery, and the recovery process is designed. Under the assumption of a small bending cable, a cable model that simultaneously considers the elasticity, mass, and aerodynamic force is established. After the analysis, when the airflow velocity is large (50m/s and above), the change in the cable configuration is mainly caused by the aerodynamic force, which becomes the main factor affecting the cable spatial configuration. In addition, increasing the cable tension can reduce the bending phenomenon of the cable. The workspace is discretized into spatial scatter points, and the CDPR error and cable-driven power consumption are analyzed point by point, with a subsequent analysis of their spatial distribution characteristics. In order to improve the comprehensive performance of the recovery system, a multi-objective optimization method is proposed, taking into account the error distribution, power consumption distribution, and safety distance. The optimized CTLL and interception space position coordinates are determined through solving with a genetic algorithm, and comparative analysis with the initial condition indicates an 83% reduction in error, a 62.3% decrease in power consumption, and a 1.2 m increase in safety distance.

The cable model established in this paper, considering the steady-state aerodynamic force, provides a simplified analytical model for the application of a non-suspended CDPR in airflow environments, which is conducive to the expansion of the application scenarios of a CDPR. The proposed CDPR-based recovery system introduces a new design concept for UAV aerial recovery systems, and the conducted analysis establishes the foundation for subsequent in-depth research.

## Figures and Tables

**Table 1:** Calculation results of relative scale values at $p_{i}$.

Configuration	v (m/s)	$p_{1}$	$p_{2}$	$p_{3}$	$p_{4}$	$p_{5}$	$p_{6}$
$C_{1}$	0	0	0	0	0	0	0
$C_{2}$	5.6	4.6%	4.6%	4.6%	4.6%	4.6%	4.6%
$C_{3}$	26.4	102.3%	102.5%	102.6%	102.7%	102.7%	102.7%
$C_{4}$	50	341.8%	345.8%	348.8%	351%	352.2%	352.6%
$C_{5}$	80	662.8%	691.6%	717.4%	737.9%	750.8%	754.9%
$C_{6}$	100	801.7%	852.4%	902.5%	946.9%	977.9%	988.4%

**Table 2:** Calculation of spatial distances of observation points under different tensions.

Configuration	$\left|t_{B}\right|$ (kN)	$p_{1}$ (m)	$p_{2}$ (m)	$p_{3}$ (m)	$p_{4}$ (m)	$p_{5}$ (m)	$p_{6}$ (m)
$C_{50}\;*$	0.5	2.87	5.41	7.53	9.13	10.12	10.45
$C_{51}$	0.7	2.32	4.31	5.92	7.11	7.84	8.08
$C_{52}$	0.9	1.92	3.54	4.83	5.77	6.33	6.52
$C_{53}$	1.3	1.4	2.56	3.48	4.13	4.53	4.66
$C_{54}$	2.1	0.89	1.63	2.2	2.61	2.85	2.94
$C_{55}$	3.7	0.51	0.93	1.26	1.49	1.63	1.67
$C_{56}$	6.9	0.27	0.5	0.67	0.8	0.87	0.9
$C_{57}$	13.3	0.14	0.26	0.35	0.41	0.45	0.47
$C_{58}$	26.1	0.07	0.13	0.18	0.21	0.23	0.24
$C_{59}$	51.7	0.04	0.07	0.1	0.12	0.13	0.14

* $C_{50}$ represents the configuration $C_{5}$.

**Table 3:** The structural and aerodynamic parameters for the CDPR.

Parameter Name	Parameter Symbol	Value
Cable diameter	D	0.01 m
Cable cross-sectional area	A	$7.854\times 10^{-5}\;m^{2}$
Young’s modulus	E	1.6 GPa
Cable density	$\rho_{c}$	$999.5\;\mathrm{kg}/m^{3}$
Span 1	$d_{1}$	4 m
Span 2	$d_{2}$	3 m
Telescopic rod weight	m	122 kg
Telescopic rod shortening length	${\left|\vec{\mathrm{OP}}\right|}_{\min}$	3.12 m
Telescopic rod elongation length	${\left|\vec{\mathrm{OP}}\right|}_{\max}$	6.22 m
Air density at 3 km altitude	$\rho_{a}$	$0.9096\;\mathrm{kg}/m^{3}$
Aerodynamic friction coefficient	$c_{t}$	0.02
Aerodynamic drag coefficient	$c_{n}$	0.8
Carrier aircraft flight speed	v	80 m/s
Gravity acceleration	g	$9.8\;m/s^{2}$

**Table 4:** Optimization results.

	CTLL (N)	$\left(c_{x},c_{y},c_{z}\right)$
Initial	1000	(4.6,0,3.1)
Optimal	500	(−3.4,0,4.3)

**Figure A1:**
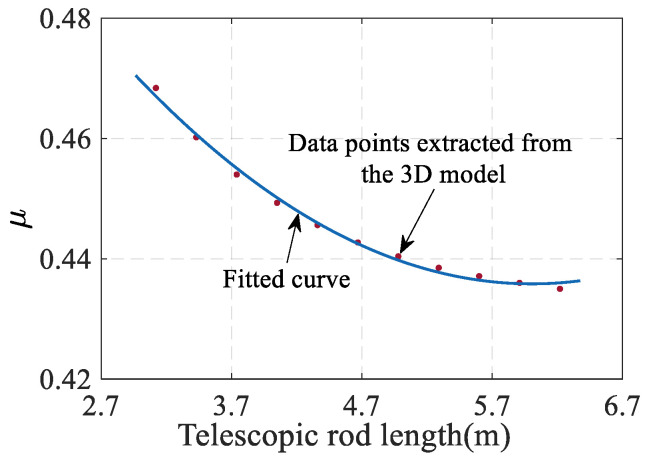
Aata fitting for scale factor μ.

**Figure A2:**
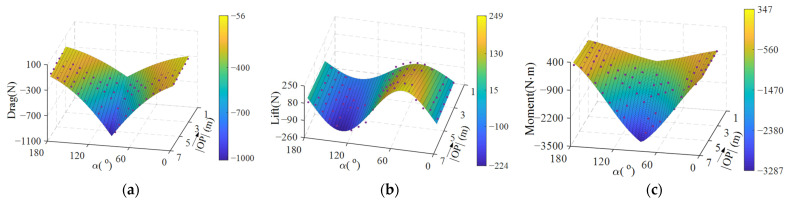
Data fitting for aerodynamic force and moment: (**a**) data fitting for aerodynamic drag; (**b**) data fitting for aerodynamic lift; (**c**) data fitting for aerodynamic moment.

## Data Availability

Data are contained within the article.

## Abbreviations

UAV	unmanned aerial vehicle;
CDPR	cable-driven parallel robot;
CTLL	cable tension lower limit;
DARPA	Defense Advanced Research Projects Agency;
MOP	multiple-objective problems;
DM	decision maker;
PF	Pareto Front;
TOPSIS	technique to order of preference by similarity to ideal solution;
GA	genetic algorithms;
PSO	particle swarm optimization;
DE	differential evolution;
ACO	ant colony optimization;
MOSA	multi-objective simulated annealing.

## Appendix A

1. Data fitting for $\vec{OP_{\mathrm{mg}}}$ and $M_{\mathrm{mg}}$

The telescopic rod has an irregular shape and uneven mass distribution, the center of the gravity position of different $\left|\vec{\mathrm{OP}}\right|$ lengths is extracted by 3D modeling, and the scale factor μ is obtained by data fitting, and the fitting expressions and curves are as follows:

(A1) $$\mu =0.003751{\left|\vec{\mathrm{OP}}\right|}^{2}-0.04504\left|\vec{\mathrm{OP}}\right|+0.5711$$

Furthermore, the expression describing the relationship between $\vec{OP_{\mathrm{mg}}}$ and $\vec{\mathrm{OP}}$ is derived.

(A2) $$\vec{OP_{\mathrm{mg}}}=(0.003751{\left|\vec{\mathrm{OP}}\right|}^{2}-0.04504\left|\vec{\mathrm{OP}}\right|+0.5711)\vec{\mathrm{OP}}$$

The expression for $M_{\mathrm{mg}}$ is obtained.

(A3) $$M_{\mathrm{mg}}=(0.003751{\left|\vec{\mathrm{OP}}\right|}^{2}-0.04504\left|\vec{\mathrm{OP}}\right|+0.5711)\vec{\mathrm{OP}}\times mg$$

2. Data fitting for $F_{w}$ and $M_{w}$

The carrier aircraft is flying straight with a speed v=80 m/s, and the airflow velocity is $V_{w}=(-80\;0\;0)\;m/s$ in the body coordinate system. α is the angle between the telescopic rod axis $\vec{\mathrm{OP}}$ and the +*x*-axis, and β is the angle between the projection vector of the telescopic rod axis $\vec{\mathrm{OP}}$ and the +*y*-axis in the yoz plane, in which $0^{\circ }<\alpha <180{}^{\circ}$, and 0°<β<180°. Firstly, α and $\left|\vec{\mathrm{OP}}\right|$ are discretized at equal intervals; secondly, the aerodynamic force and moment of the telescopic rod under the combined conditions of α and $\left|\vec{\mathrm{OP}}\right|$ are calculated by the finite element; finally, $F_{w}$ and $M_{w}$ are obtained through data fitting. It is necessary to note that the value of β in the above calculation process is always β=90°, and we can multiply the trigonometric function of β when β≠90°, thus saving a lot of time in the finite element analysis. $F_{w}$ and $M_{w}$ are functions of α, β, and rod length $\left|\vec{\mathrm{OP}}\right|$, and the fitted expression and graph are as follows:

(A4) $$F_{w}=\left(\begin{array}{c} (-500\cos(0.0063\left|\alpha -90\right|+2.356)-512.7)\left|\vec{\mathrm{OP}}\right|\\ \left(-37.19\sin(0.03715\;\alpha +2.938\;)\left|\vec{\mathrm{OP}}\right|+12.82)\cos(\beta \right)\\ \left(-37.19\sin(0.03715\;\alpha +2.938\;)\left|\vec{\mathrm{OP}}\right|+12.82)\sin(\beta \right) \end{array}\right)$$

(A5) $$M_{w}=\left(\begin{array}{c} 0\\ \left(-50\cos(0.0216\left|\alpha -84.99\right|+0.5256)-38.13)){\left|\vec{\mathrm{OP}}\right|}^{2}\sin(\beta \right)\\ \left(-50\cos(0.0216\left|\alpha -84.99\right|+0.5256)-38.13)){\left|\vec{\mathrm{OP}}\right|}^{2}\cos(\beta \right) \end{array}\right)$$
